# 2D Janus ZrSSe/SnSSe Heterostructure: A Promising Candidate for Photocatalytic Water Splitting

**DOI:** 10.1021/acsomega.3c08620

**Published:** 2024-04-24

**Authors:** Nabeel Anjum, Muhammad Kashif, Aamir Shahzad, Abdur Rasheed, Guogang Ren

**Affiliations:** † Physics Department, Govt. College University Faisalabad (GCUF), Allama Iqbal Road, Faisalabad 38000, Pakistan; ‡ School of Physics, Engineering and Computer Science, University of Hertfordshire, Hatfield AL10 9AB, U.K.

## Abstract

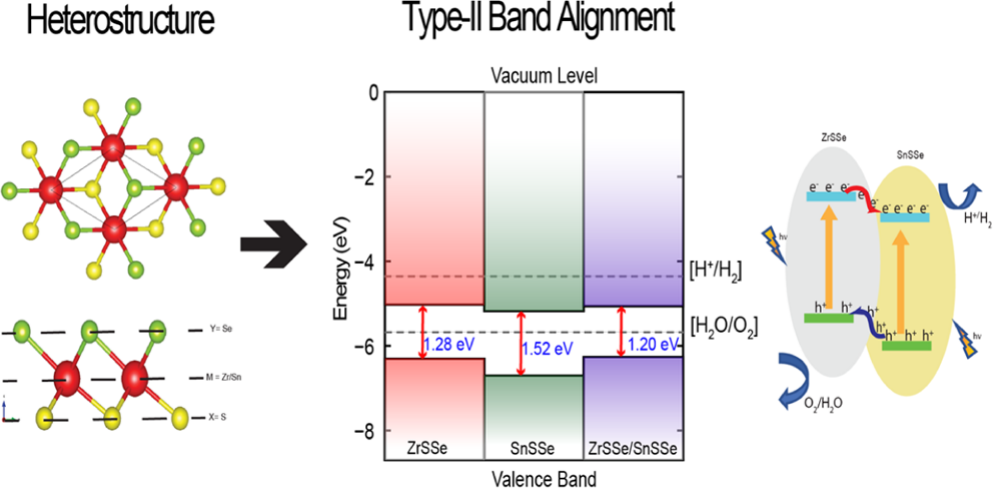

The distinctive physical characteristics and wide range of potential applications in optoelectronic and photovoltaic devices have ignited significant interest in two-dimensional materials. Intensive research attention has been focused on Janus transition metal dichalcogenides due to their unique properties resulting from symmetry disruption and their potential in photocatalysis applications. Motivated by the current fascination with Janus TMD heterostructures, we conducted first-principles calculations to examine the stability, electronic, and optical properties of monolayers consisting of ZrSSe, SnSSe, and the ZrSSe/SnSSe heterostructure. The results indicate that the Janus ZrSSe/SnSSe heterostructure exhibits a structural and mechanical stability. Using the HSE06 functional, the ZrSSe/SnSSe heterostructure shows an indirect band gap of 1.20 eV, and band edge analysis reveals a type-II band alignment. The potential for photo/electrocatalysis in the ZrSSe/SnSSe heterostructure for water splitting or generating reactive oxygen species (ROS) has been explored, and it was found that the oxygen evolution reaction (OER) can spontaneously activate in acidic (pH = 0) media under light irradiation, with a potential of *U* = 1.82 eV. Additionally, the ZrSSe/SnSSe heterostructure exhibits strong light absorption across a wide range, from visible light to the ultraviolet region, at various levels. These findings open up possibilities for the application of ZrSSe/SnSSe-based materials in optoelectronic devices.

## 1 Introduction

Two-dimensional (2D) materials, particularly transition metal dichalcogenides (TMDs) like MoS_2_, MoSe_2_, WS_2_, WSe_2_, and ZrS_2_, have gained significant attention in scientific research due to their remarkable properties arising from reduced dimensionality.^1−3^ These properties, including exceptional electrical, optical, mechanical, and thermal characteristics, have led to their wide-ranging applications in catalysts, batteries, and solar cells.

Recent research efforts, both theoretical and experimental, have been dedicated to 2D materials-based van der Waals (vdW) heterostructures. These heterostructures involve stacking distinct monolayers vertically, resulting in properties superior to those of individual monolayers. For example, graphene/h-BN heterostructures have the potential to revolutionize semiconductor device applications,^4^ and MoS_2_/WS_2_ heterostructures exhibit rapid charge transfer.^5^ Various such heterostructures, such as graphene combined with transition metal dichalcogenides (TMDs) and CdO with GaS, exhibit intriguing properties.^6,7^ VdW heterostructures can be classified into three primary types of band alignments, straddling type-I, staggered type-II, and broken-gap type-III, offering diverse applications, including LEDs, quantum well lasers, field-effect transistors (FETs), photocatalysts, and photovoltaics.

Traditionally, TMD monolayers have two chalcogen layers of the same element. However, if these layers contain different chalcogen atoms, then a Janus monolayer forms, reducing overall symmetry. The initial Janus transition metal dichalcogenide (TMD), MoSSe, was successfully synthesized through the chemical vapor deposition (CVD) method.^8,9^ This same approach has been applied to the synthesis of MoSSe by using CVD as well. Furthermore, a more recent development involves a room-temperature technique for the synthesis of various high-quality Janus TMDs, including MoSSe and WSSe.^10^ This method relies on a selective epitaxy atomic replacement process, leveraging precursors to efficiently remove and replace the top chalcogen layer. Recent theoretical investigations have explored the unique structural, thermal, magnetic, electronic, and optoelectronic properties of Janus transition metal dichalcogenides (JTMDs).^11^ Nonetheless, both experimental and theoretical research on JTMDs are in their early stages, requiring further theoretical understanding to assess their performance accurately in diverse applications.

Theoretical investigations have uncovered diverse potential applications for Janus monolayers across various domains. These applications include their utility as materials for structural phase transitions,^12^ facilitating photocatalytic water splitting^13^ and generating concurrent in-plane/out-of-plane piezoelectricity.^14^ Moreover, when these Janus monolayers are incorporated into multilayer structures or combined with other 2D materials, they may induce intriguing effects. For instance, their substantial intrinsic dipole moment can aid in separating intralayer excitons into interlayer excitons,^15^ enable the movement of charges between different layers within a heterostructure,^16,17^ or provide opportunities for precise control of band alignment and Schottky barriers at interfaces. These findings highlight the versatility and potential of Janus monolayers in the advancement of various technological applications.

Researchers such as Wang et al.^18^ have conducted systematic analyses on another group of Janus monolayers denoted as MXY (with M = Mo, W; X/Y = S, Se, Te; and X ≠ Y). Their investigations revealed that WSSe and WSeTe monolayers exhibited superior carrier mobility compared to MoS_2_. Additionally, Huang et al.,^19^ employed DFT calculations to explore the stabilities, electronic properties, and optical characteristics of Janus group III monochalcogenide monolayers, labeled as M_2_XY (where M = Ga and In, and X/Y = S, Se, and Te). Their results indicated high absorption coefficients (∼3 × 10^4^) in the visible light range for these monolayers, all of which displayed semiconductor characteristics. Moreover, Cui et al.^20^ conducted research focusing on Janus MoSSe and ZnO van der Waals heterostructures. This work unveiled variable bandgaps ranging from 0.31 to 0.91 eV when exposed to a vertical electric field, with distinct peaks within the visible light spectrum. ZrSSe and SnSSe, two compounds belonging to the transition metal chalcogenide family, have garnered significant attention. First-principles calculations have indicated that monolayer SnSSe exhibits higher hole and electron mobilities in comparison to SnS_2_ and SnSe_2_ monolayers.^21^ Furthermore, the Janus ZrSSe monolayer possesses a band gap of approximately 1.341 eV, rendering it capable of absorbing both visible and ultraviolet light ranges.^22^ These unique properties and prospective applications of Janus ZrSSe and SnSSe monolayers make them compelling materials for researchers and engineers working in the fields of electronics and optoelectronics.

In this study, we conducted a systematic investigation of the structural, electronic, optical, and photocatalytic properties of the ZrSSe/SnSSe heterostructure through first-principles analysis. Our findings reveal that the ZrSSe/SnSSe heterostructure exhibits thermodynamic stability, possesses a band gap of 1.20 eV, demonstrates favorable band edge alignments, and offers a sufficient driving force for water splitting through the OER process. Additionally, the ZrSSe/SnSSe heterostructure exhibits notable absorption in the visible range of the solar spectrum, suggesting its potential as a highly promising photocatalyst for direct water splitting.

## 2 Computational Methodology

Density functional theory (DFT) calculations^23^ were conducted employing the Vienna Ab-initio Simulation Package (VASP).^24^ Projector-augmented wave (PAW) potentials^25^ and the generalized gradient approximation (GGA) within the Perdew–Burke–Ernzerhof (PBE) framework were utilized to describe the exchange-correlation potential.^26^ To account for van der Waals interactions, Grimme’s DFT-D2 correction method was applied.^27^ A cutoff energy of 500 eV was chosen for the plane wave basis, while energy and force convergence criteria were set to 10^–6^ eV and 0.01 eV/Å, respectively. Monkhorst–Pack sampling^28^ with a 35 × 35 × 1 k-point grid was used to represent the Brillouin zone in relaxation and other calculations. For precise band structure analysis, the Heyd–Scuseria–Ernzerhof (HSE06) hybrid functional,^29^ incorporating 25% Hartree–Fock exchange energy, was employed.^30^ Additionally, a 20 Å vacuum layer was introduced in the *z*-direction to account for interlayer interactions

## 3 Results and Discussion

### 3.1 Structure and Stability

**Figure 1:**
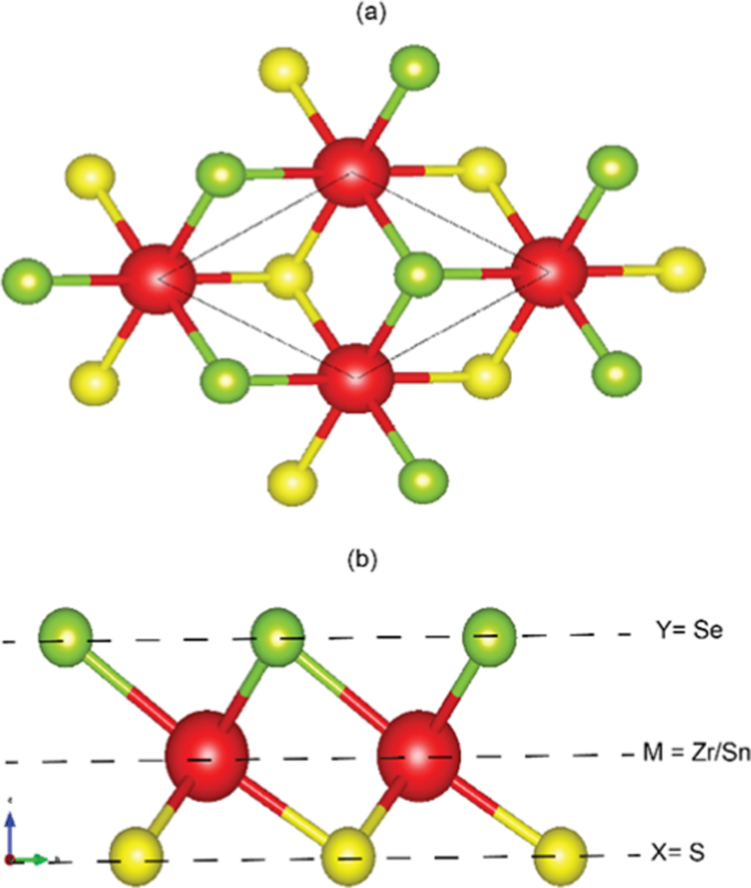
(a) Top and (b) side views of Janus Monolayer.

**Figure 2:**
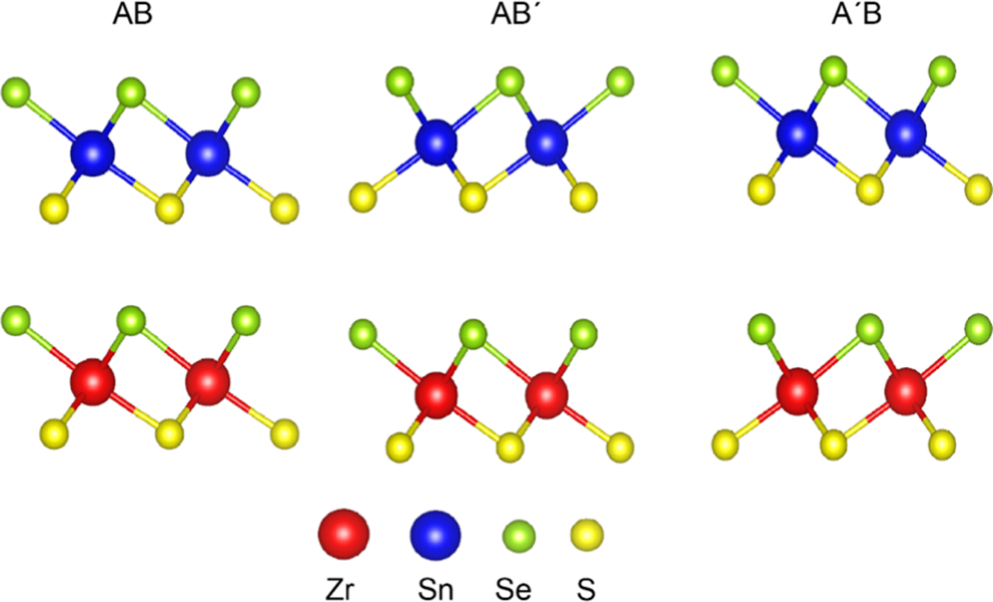
ZrSSe/SnSSe heterostructure with three different stacking patterns AB, A′B, and AB′ where the red, blue, green, and yellow colors represent Zr, Sn, Se, and S atoms, respectively.

The transition metal dichalcogenides (TMDs), specifically MX_2_ (where M = Zr, Sn; X = S, Se), exhibit semiconducting characteristics in their 1T-phase.^31^ These structures adopt a 1T-CdI_2_-like configuration with the *P*3̅*m*1 space group, where the M atom is situated between two chalcogen atoms (X = S, Se), forming a single atomic layer within the trigonal structure.^32^ In bulk TMDs, a layered structure is observed where each layer mirrors its adjacent layer, and weak van der Waals interactions prevail between the layers. In the context of Janus monolayers, denoted as MXY (where M = Zr, Sn, and X ≠ Y; X/Y = S, Se), these structures are constructed using two dissimilar chalcogen atoms, X and Y (X ≠ Y). As depicted in Figure 1, analogous to pristine MX_2_, Janus MXY monolayers also adopt BiTeI-like structures, maintaining the *P*3*m*1 space group.^33^ Consequently, this work focuses on two distinct Janus MXY monolayers, namely, ZrSSe and SnSSe. These Janus MXY monolayers are combined to form a heterostructure, illustrated in the Figure 2.

Initially, we determined the optimized lattice parameters for both the monolayers and the heterostructure. Following this, a stable configuration for the heterostructure was chosen for subsequent calculations. The optimized lattice constants for the ZrSSe and SnSSe Janus monolayers were found to be 3.72 and 3.76 Å, respectively, demonstrating excellent agreement with values previously reported by researchers.^21,22^

For constructing the ZrSSe/SnSSe van der Waals heterostructures (vdWHs), we utilized 1 × 1 monolayers of ZrSSe and SnSSe. The lattice mismatch between these monolayers was calculated to be 1.06%. To identify the most suitable stable configuration, we explored three potential stacking patterns for the ZrSSe/SnSSe heterostructure. These configurations, denoted as AB, A’B, and AB’, are illustrated in Figure 2. Here, A represents the ZrSSe monolayer, B represents the SnSSe monolayer, and A’ and B’ correspond to the monolayers rotated 180° relative to each other.

To determine the most stable structure of the heterostructures while varying the interlayer spacing, we employ the binding energy as a crucial metric. The binding energy is calculated by using the following equation
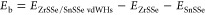
1Where *E*_ZrSSe/SnSSe vdWHs_, *E*_ZrSSe_, and *E*_SnSSe_ are the total energies of ZrSSe/SnSSe heterostructure, SnSSe monolayer, and ZrSSe monolayer, respectively.

**Figure 3:**
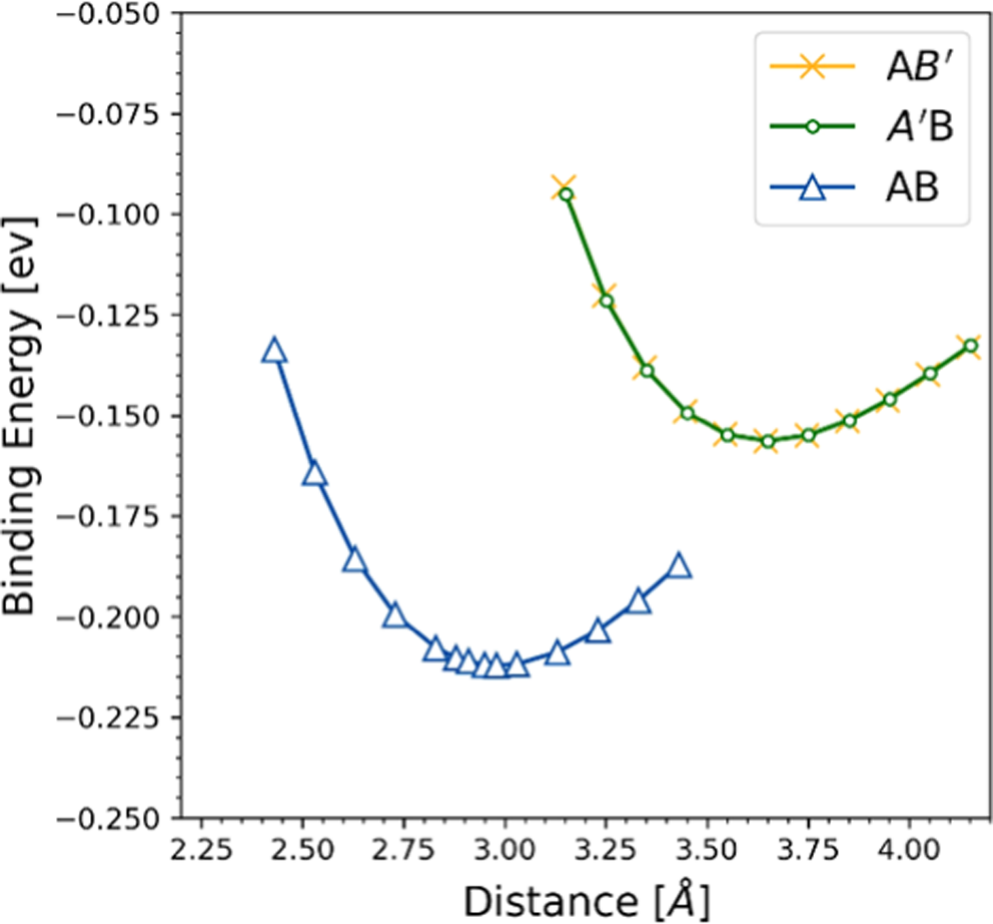
Binding energy of heterostructures with varied interlayer distance.

The binding energy’s dependence on the interlayer distance is presented in Figure 3. For the AB structure, the equilibrium interlayer distance is determined to be 2.98 Å, which corresponds to the lowest binding energy of about −0.21 eV. this binding energy of heterostructure is the same as the binding energy of other 2D vdW heterostructures, such as MoS_2_/PbI_2_ (−0.228 eV),^34^ SnS_2_/PbI_2_ (−0.124 eV),^35^ SiC/GaN (−0.375 eV).^36^

In contrast, for the A’B and AB’ configurations, the equilibrium interlayer distance is nearly identical, approximately 3.64 Å. It is important to note that a negative binding energy signifies the stability of the obtained heterostructure.

### 3.2 Mechanical Properties

We conducted a comprehensive examination of the mechanical properties of the ZrSSe/SnSSe heterostructure as well as the individual ZrSSe and SnSSe monolayers. In the context of 2D materials, a critical aspect involves the study of their elastic constants, which play a significant role in assessing their mechanical behavior. These structures possess four primary independent elastic stiffness constants: *C*_11_, *C*_22_, *C*_12_, and *C*_66_. Hexagonal lattice structures, owing to their inherent symmetry, exhibit specific relationships, notably, *C*_11_ = *C*_22_ and *C*_66_ = 1/2(*C*_11_–*C*_12_). *C*_11_ and *C*_22_ represent the material’s stiffness response when subjected to uniaxial strains along the *x* and *y* directions, respectively. *C*_12_ characterizes the response to a biaxial strain state, while *C*_66_ reflects the material’s capacity to resist in-plane shear strain. The calculated values of elastic constants (*C*_ij_), Young’s modulus (*Y*_2D_), and Poisson’s ratio (ν) for the ZrSSe/SnSSe heterostructure, the ZrSSe monolayer, and the SnSSe monolayer are detailed in Table 1. The calculated elastic constants affirm the fulfillment of the Born-Huang mechanical stability criteria for 2D hexagonal,^37^ i.e., *C*_11_, *C*_22_, *C*_66_ > 0 and *C*_11_*C*_22_ – *C*_12_^2^ > 0. Consequently, both the heterostructure and monolayers are deemed mechanically stable. Using these elastic constants, we derived additional mechanical properties, including the 2D layer modulus (γ^2D^), Young’s modulus (*Y*_2D_), shear modulus (*G*), and intrinsic strength (σ_int_), using the given formulas.
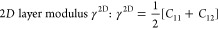
2
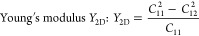
3
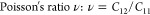
4
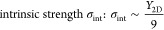
5

**Table 1:** Calculated Values for Elastic Modulus Tensor *C*_ij_ (Nm^–1^), 2D Young’s Modulus (Nm^–1^), Poisson’s Ratios ν_2D_, 2D Layer Modulus (Nm^–1^), and Intrinsic Strength (Nm^–1^)

	*C*_11_	*C*_12_	*G*	*Y*_2D_	ν_2D_	γ^2D^	σ_int_	status
ZrSSe	97	31	32	87	0.32	64	9.66	stable
SnSSe	77	26	25	68	0.33	51.5	7.55	stable
ZrSSe/SnSSe	138	30	53	131	0.22	84	14.55	stable

**Figure 4:**
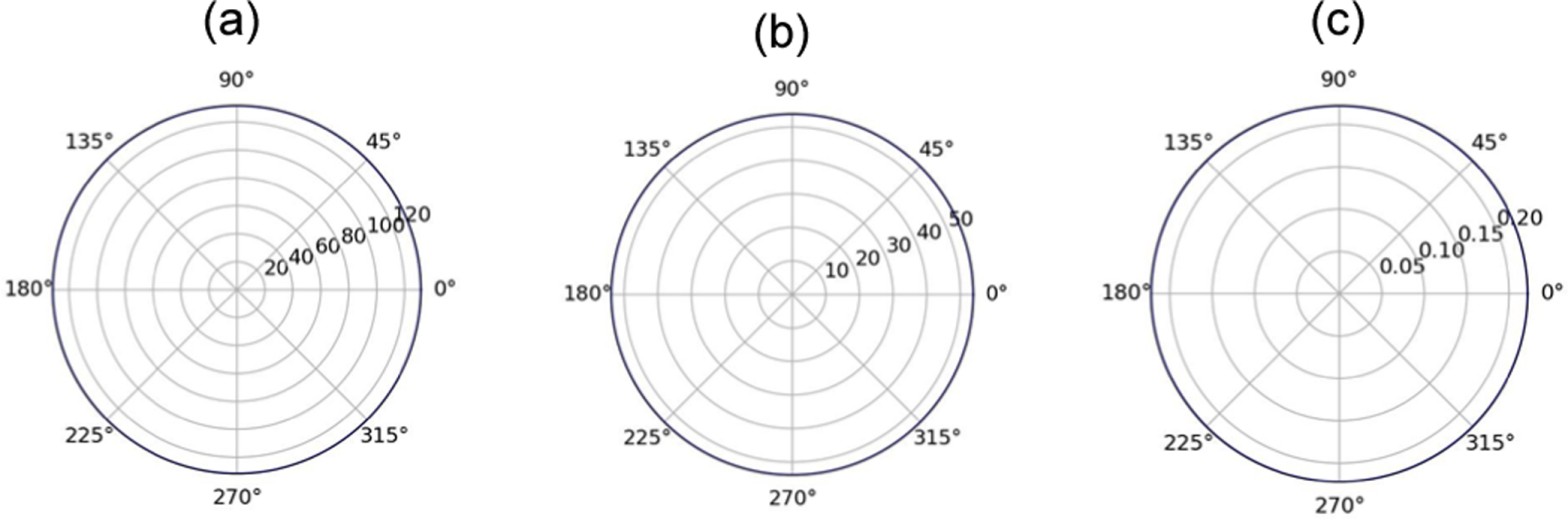
2D polar plot of (a) Young’s modulus, (b) shear modulus, and (c) Poisson’s ratio of the ZrSSe/SnSSe heterostructure.

The results indicate a substantial increase in Young’s modulus and the 2D layer modulus of the ZrSSe/SnSSe heterostructure, with enhancements of 48 and 38% compared to the individual monolayers, respectively. Young’s modulus reflects a material’s resistance to deformation under stress, while the 2D layer modulus signifies its resistance to stretching. This suggests that the ZrSSe/SnSSe heterostructure exhibits greater stiffness than the ZrSSe and SnSSe monolayers. Furthermore, the results imply that both the ZrSSe and the SnSSe monolayers exhibit mechanical isotropy. In the case of the ZrSSe/SnSSe heterostructure, the small difference between *C*_11_ and *C*_12_ leads to the relationship *C*_11_ – *C*_12_ = 2*C*_66_, demonstrating the isotropic behavior of the heterostructure, as depicted in Figure 4(a–c).

In the heterostructure, the binding strength between the two monolayers is notably enhanced, owing to the strong interlayer interactions. This increased binding strength results in a reduction of Poisson’s ratio, as there is minimal transverse strain under external stress. Furthermore, the shear modulus and intrinsic strength of the heterostructure exhibit significant enhancements compared to the individual monolayers. The shear modulus is indicative of how 2D materials can resist folding and rippling, thereby influencing the scattering behavior of charge carriers within the structure.

The findings indicate that both the ZrSSe and SnSSe monolayers exhibit good mechanical stability, characterized by high Young’s modulus values and excellent resistance to deformation. When combined into the ZrSSe/SnSSe heterostructure, it is anticipated that these mechanical attributes will be influenced by interlayer interactions, potentially augmenting the overall stiffness and resistance to deformation of the heterostructure. This comprehensive assessment underscores the significance of comprehending the mechanical characteristics of these materials, which is imperative for their effective utilization across diverse technological domains. The analysis of these elastic constants furnishes invaluable insight into the mechanical traits of 2D materials and their heterostructures. A thorough comprehension of their mechanical stability and stiffness is a pivotal requirement for the successful integration of these materials in various applications.

### 3.3 Electronic Properties

**Figure 5:**
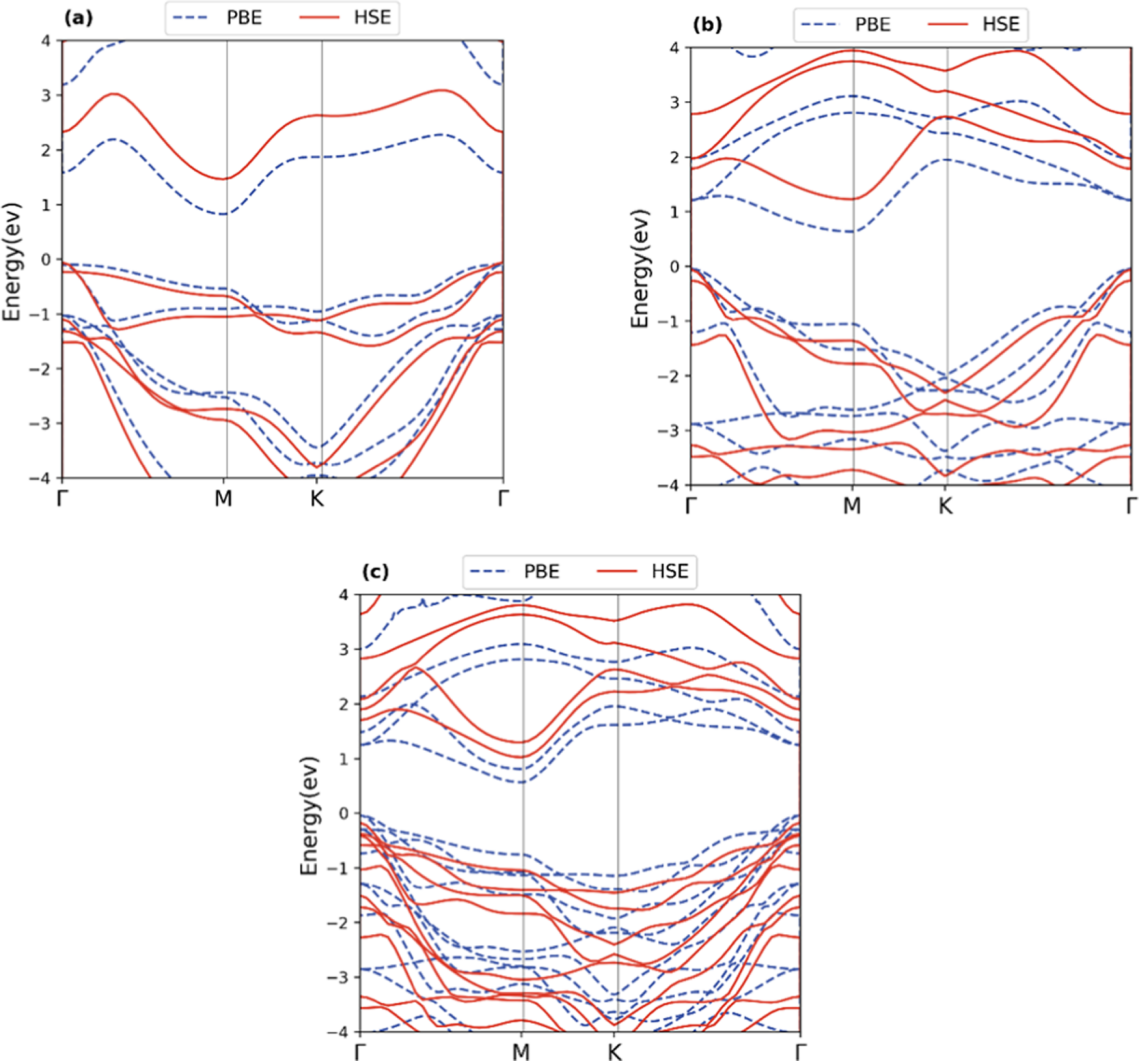
Band structure of (a) SnSSe, (b) ZrSSe, and (c) ZrSSe/SnSSe heterostructure.

We conducted an in-depth examination of the electronic properties of both the monolayers and the heterostructure through an analysis of the electronic band structures and density of states (DOS). The energy band structures of the individual Janus monolayers, namely, ZrSSe and SnSSe, as well as the ZrSSe/SnSSe heterostructure, were examined using the GGA-PBE and HSE06 methods, as depicted in Figure 5. Notably, the band structures obtained from both methods exhibit the same shape and behavior.

**Figure 6:**
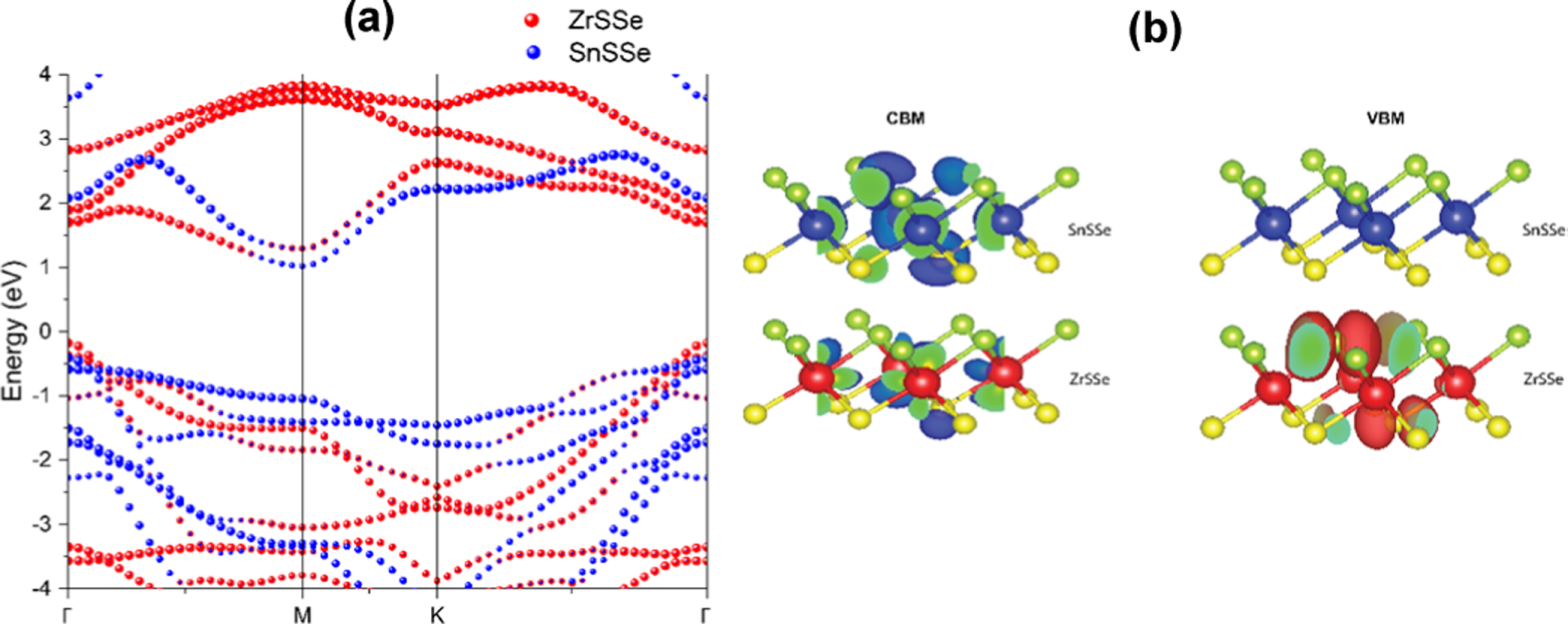
(a) Projected band structure of ZrSSe/SnSSe heterostructure. (b) Band decomposed charge density of VBM and CBM.

Specifically, the ZrSSe monolayer shows an indirect band gap of 1.28 eV, situated between the Γ and M points of the Brillouin Zone, as illustrated in Figure 5(a). This result agrees with a prior DFT study employing the HSE06 method (band gap = 1.34 eV)^22^ and the experimental band gap ZrSSe monolayer is 1.32 eV.^38^ Similarly, the SnSSe monolayer exhibits an indirect band gap of 1.52 eV, as demonstrated in Figure 5(b). The band structure of the ZrSSe/SnSSe heterostructure is shown in Figure 5(c), revealing an indirect band gap of 1.20 eV. Notably, this band gap value is lower than that of both the Janus ZrSSe and SnSSe monolayers. In the ZrSSe/SnSSe heterostructure, the conduction band minimum (CBM) is situated at the M point, while the valence band maximum (VBM) is located at the Γ point. It is worth noting that the heterostructure exhibits an indirect band gap with a magnitude of 1.20 eV. Furthermore, the projected band structure of the ZrSSe/SnSSe heterostructure, illustrated in Figure 6(a), elucidates that the CBM primarily arises from the SnSSe monolayer, whereas the VBM predominantly originates from the ZrSSe monolayer. This observation is corroborated by the band-decomposed charge densities, as depicted in Figure 6(b). Consequently, it can be inferred that the VBM and CBM of the heterostructure originate from distinct layers, indicative of a type-II band alignment. This particular band alignment holds promise for the spontaneous separation of electron–hole pairs, rendering it valuable for applications in solar energy conversion and photocatalysis.

**Figure 7:**
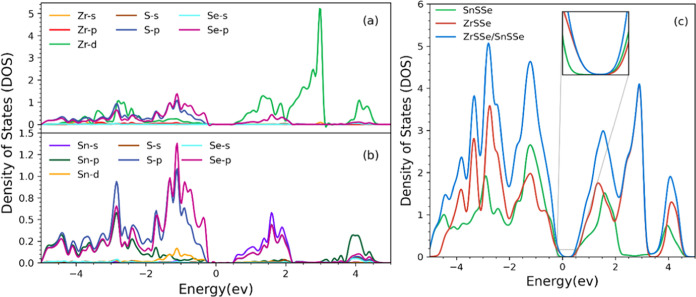
(a, b) PDOS of ZrSSe/SnSSe heterostructure. (c) Total density of states of ZrSSe/SnSSe heterostructure.

In addition to analyzing the band structure, we performed calculations for the total density of states (TDOS) and projected density of states (PDOS) of the ZrSSe/SnSSe heterostructure, presented in Figure 7(a). The PDOS analysis of the ZrSSe/SnSSe heterostructure reveals that the valence band maximum (VBM) predominantly arises from the contributions of the S-p and Se-p orbitals, while the conduction band minimum (CBM) exhibits nearly equal contributions from the Zr-d, Sn-s, and Se-p orbitals, as depicted in Figure 7(b). Furthermore, an examination of the total DOS for both the heterostructure and the individual monolayers demonstrates that the CBM of the ZrSSe/SnSSe heterostructure predominantly originates from the SnSSe monolayer, while the VBM is primarily derived from the ZrSSe monolayer, as highlighted in Figure 7(c) (inset).

### 3.4 Charge Density Difference

**Figure 8:**
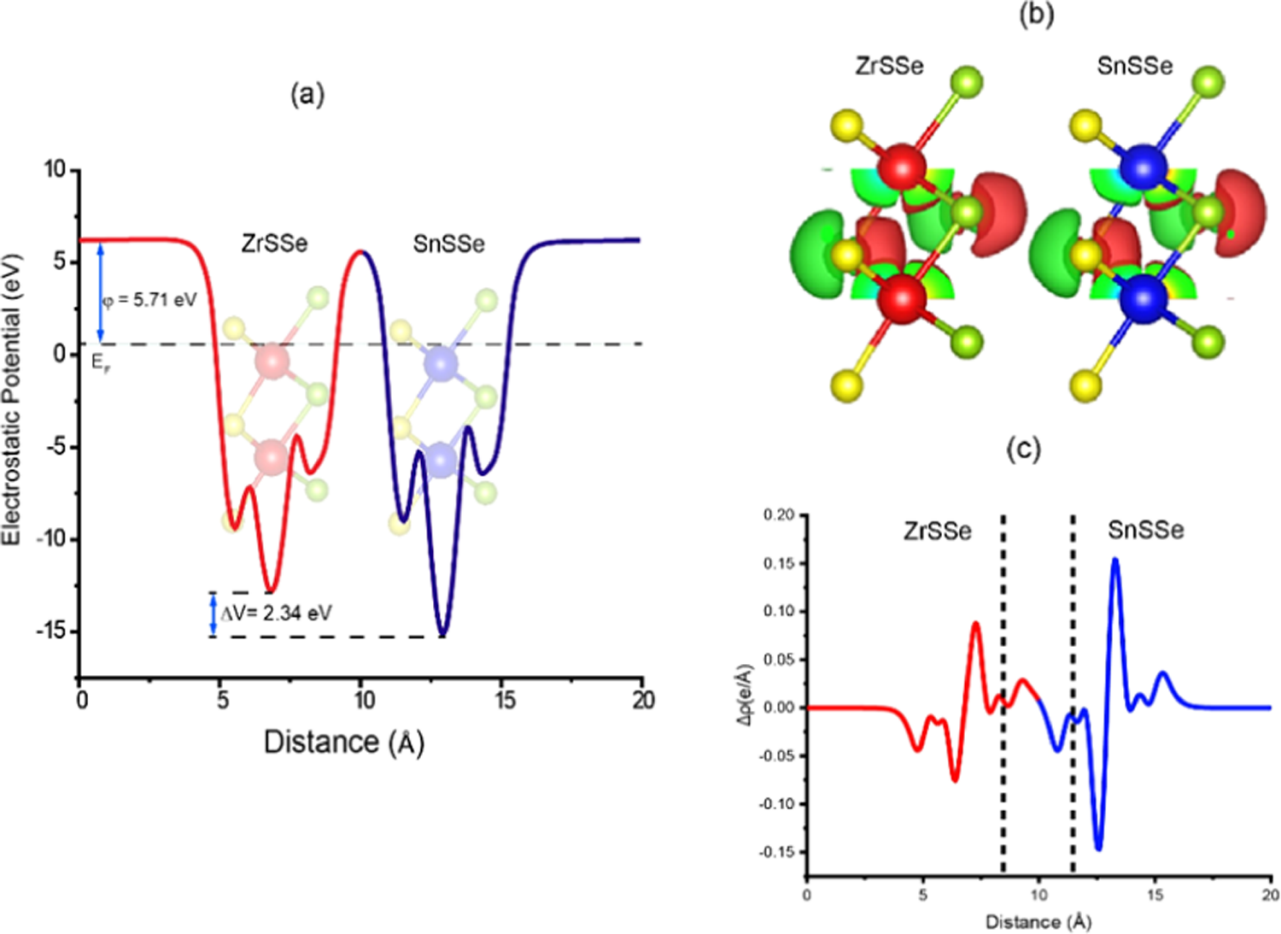
(a) Electrostatic potential of the ZrSSe/SnSSe heterostructure along the *z*-direction and (b) charge density difference of the ZrSSe/SnSSe heterostructure; green and red colors correspond to the accumulation and depletion of electronic densities. (c) Charge density difference in 1-D.

To investigate the charge separation at the ZrSSe/SnSSe heterojunction, we conducted a 3D charge density difference analysis for the heterostructure. The charge density difference plot is presented in Figure 8(c), and the calculation of charge density difference between the layers was based on the following equation
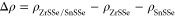
6where ρ_ZrSSe/SnSSe_, ρ_ZrSSe_, and ρ_SnSSe_ represent the charge density of ZrSSe/SnSSe heterostructure, charge density of the ZrSSe and SnSSe monolayer, respectively.

Figure 8(b) reveals areas of electron depletion represented in red and electron accumulation depicted in green, indicating charge redistribution between the heterostructure layers. Specifically, there is charge depletion in the ZrSSe layer, accompanied by charge accumulation in the SnSSe layer, resulting in a built-in electric field within the monolayers. The planar-averaged charge density difference along the *z*-direction, as shown in Figure 8(c), demonstrates positive values signifying charge accumulation and negative values indicating charge depletion. These findings are consistent with Bader charge analysis, confirming a charge of 0.0125e transfer from the ZrSSe layer to the SnSSe layer. This charge transfer establishes a built-in electric field between the layers, which facilitates electron separation.

The electrostatic potential of the ZrSSe/SnSSe heterostructure along the *z*-direction at equilibrium is depicted in Figure 8(a). Notably, the SnSSe monolayer exhibits a more negative potential compared to the ZrSSe monolayer, resulting in a potential difference of approximately 2.34 eV between the heterostructure layers. This potential difference generates a strong built-in electric field between the layers, reducing the recombination rate of photogenerated electron–hole pairs and showcasing potential applications in optoelectronic devices.

The work functions of ZrSSe, SnSSe, and the ZrSSe/SnSSe heterostructure are determined to be −5.67, −6.19, and −5.71 eV, respectively. Work functions were computed using the equation
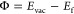
7where *E*_vac_ represents the electrostatic potential of the vacuum level and *E*_f_ denotes the Fermi level. The lower work function of the SnSSe monolayer compared to the ZrSSe monolayer implies that upon interaction to form a heterojunction, there will be charge transfer between the layers until their Fermi energy levels equilibrate. Specifically, electrons transfer from the ZrSSe layer to the SnSSe layer. After heterojunction formation, the work function of the ZrSSe/SnSSe heterojunction is reduced to −5.71 eV, as presented in Figure 8(a). This reduction is attributed to charge transfer and Fermi energy realignment to reach equilibrium. The built-in electric field resulting from this process enhances carrier mobility, reduces carrier recombination, and improves the photocatalytic properties of the heterostructure by increasing the efficiency of photoinduced electron–hole pair separation.

### 3.5 Band Edge Alignment

**Figure 9:**
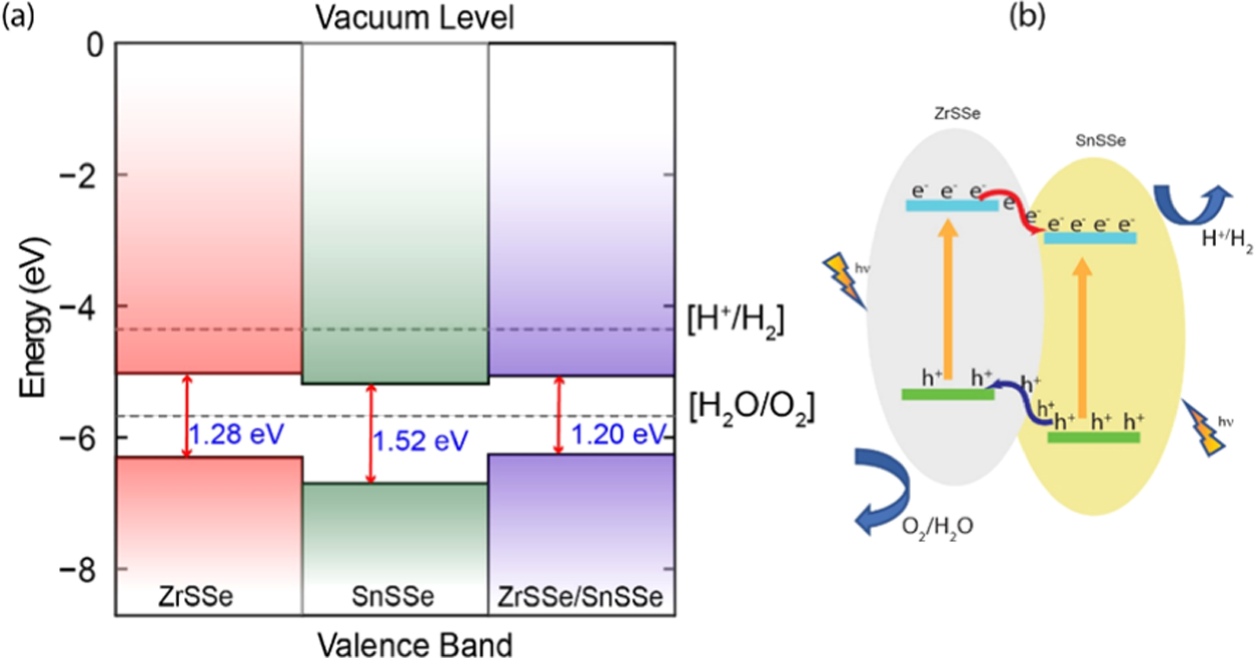
(a) Band Alignment with respect to vacuum level. (b) Schematic representation of the charge transfer path.

The determination of semiconductor band edge positions provides crucial insights into the redox capabilities and photocatalytic performance. Figure 9(a) illustrates the band alignment of the ZrSSe/SnSSe heterostructure in comparison with the isolated ZrSSe and SnSSe monolayers. Notably, the valence band maximum (VBM) and conduction band minimum (CBM) of the ZrSSe monolayer are positioned at higher energy levels than those of the SnSSe monolayer within the heterostructure, as depicted in Figure 9(a). This configuration results in staggered bands, indicative of a type-II band alignment.

Due to the energy difference between the bands, electrons within the ZrSSe monolayer undergo a transfer to the SnSSe monolayer, whereas holes migrate from the SnSSe layer to the ZrSSe layer, as shown in Figure 9(b). Consequently, the SnSSe layer becomes negatively charged, while the ZrSSe layer acquires a positive charge. This establishes an interlayer electric field directing from the ZrSSe monolayer to the SnSSe monolayer. As a result, photogenerated carriers are facilitated in transferring between the layers through this electric field. Hence, the ZrSSe/SnSSe heterostructure exhibits a type-II band alignment.

This type-II band alignment in the ZrSSe/SnSSe heterostructure restricts the photogenerated electrons and holes into different layers due to the built-in electric field, effectively restraining their recombination for extended durations. Additionally, the calculated conduction band offset (CBO) and valence band offset (VBO) between the ZrSSe and SnSSe monolayers are found to be 0.15 and 0.4 eV, respectively. The larger band offsets lead to an extended lifetime of interlayer photogenerated carriers, facilitating a more efficient carrier separation. The substantial CBO and VBO generate ample driving forces for electron and hole transitions between layers, resulting in the accumulation of photogenerated electrons and holes in separate monolayers within the ZrSSe/SnSSe heterostructure. Consequently, the prolonged lifetime of interlayer excitons contributes to a reduced carrier recombination.

Furthermore, we evaluated the photocatalytic water-splitting activity of the ZrSSe/SnSSe heterostructure by comparing the band edge potentials of ZrSSe and SnSSe to the oxidation and reduction potentials. Notably, the VBM potential of the ZrSSe/SnSSe heterostructure is lower than the oxidation potential of O^+^/H_2_O, while the CBM potential is not higher than the reduction potential of H^+^/H_2_ for the ZrSSe/SnSSe heterostructure. This analysis indicates that the ZrSSe/SnSSe heterostructure is primarily suited for the oxygen evolution reaction (OER). In comparing the oxygen evolution reaction (OER) activity between ZrSSe and SnSSe monolayers and ZrSSe/SnSSe heterostructure, both monolayers have higher valence band maximum (VBM) position relative to the oxidation potential as compared to lower VBM position of heterostructure; therefore, higher OER activity can be observed by the heterostructure. This is because electrons in the valence band have higher energy levels and are more readily available for participation in the water oxidation process. On the other hand, the monolayers with a higher VBM may experience a less efficient electron transfer, potentially leading to lower OER activity. The comparison highlights the significance of the band edge position of valence bands in influencing the catalytic performance of materials for water-splitting applications.

### 3.6 Carrier Mobility

The mobility of charge carriers plays a crucial role in facilitating the swift movement of photogenerated carriers to active sites, and it also serves as a representation of a semiconductor material’s electrical conductivity. Typically, carrier mobilities for both electrons and holes are determined by evaluating band extrema, such as the conduction band minimum (CBM) and valence band maximum (VBM). To calculate the mobilities of electrons and holes within the orthorhombic cells of ZrSSe monolayers, SnSSe monolayers, and the ZrSSe/SnSSe heterostructure, we used a recently proposed formula for anisotropic systems.^39−41^ This approach allowed us to assess carrier mobility along the Γ–*x* and Γ–*y* directions. Carrier mobility for 2D materials can be determined by using this equation
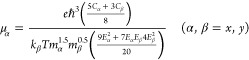
8

Where *e*, , *k*_β_, and *T* are the electron charge, reduced Plank constant, Boltzmann constant, and room temperature (300 K). *C* is the in-plane stretching modulus, which can be calculated as
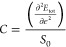
9Where *E*_tot_ is the total energy and *S*_0_ is the area.

*E*_l_ is the deformation potential constant, which can be calculated as
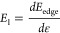
10Where *E*_edge_ is the energy of the band edge and *E* is the uniaxial strain.

Using band structure, the effective mass of the electron and holes can be calculated by fitting of parabolic functions to the VBM and CBM
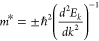
11Where *k* is the wave vector, while *E_k_* is the energy related to the wave vector *k*.

The calculated mobilities are listed in Table 2 using calculated effective masses of electron and holes, deformation potential, elastic modulus C.

**Table 2:** Effective Mass (m*), Deformational Potential Constant, Elastic Modulus, and Electron/Hole Mobility (μ) along *x* and *y* Directions of ZrSSe, SnSSe, and ZrSSe/SnSSe Heterostructure

		effective mass (m*)		deformation potential (eV)		elastic modulus (N/m)		mobility (cm^2^/V s)	
carrier type		*m_x_*	*m_y_*	*E_x_*	*E_y_*	*C_x_*	*C_y_*	μ_*x*_	μ_*y*_
electron	ZrSSe	0.238	2.251	2.014	1.414	123.63	123.61	4701	591
	SnSSe	0.230	0.754	7.15	4.858	113.98	114.45	637	235
	ZrSSe/SnSSe	0.258	0.940	6.57	5.37	218.56	237.32	1009	312
hole	ZrSSe	0.340	0.342	4.57	4.516	123.63	123.61	1099	1099
	SnSSe	0.181	0.180	9.37	9.23	113.98	114.45	859	871
	ZrSSe/SnSSe	0.358	0.358	9.688	9.61	218.56	237.32	402	412

The results reveal that both monolayers and the ZrSSe/SnSSe heterostructure exhibit anisotropic carrier mobility in the *x* and *y* directions. In particular, the electron’s effective mass in the Γ–*y* direction is slightly greater than in the Γ–*x* direction, whereas the hole’s effective mass remains relatively consistent in both directions. The results in Table 2 clearly show that the electron mobility in the *x*-direction is higher compared to the hole mobility in the *x*-direction, while the hole mobility is higher in the *y*-direction. This observation confirms the migration of electrons in the *x*-direction and holes in the *y*-direction, supporting the assertion that this behavior reduces the recombination rate of electron–hole pairs. These mobilities are still comparable with those of other 2D semiconductors, such as phosphorene (μ_h_ = 2370 and μ_e_ = 690 cm^2^/(V s))^40^ and MoS_2_ (μ_h_ = 47 and μ_e_ = 25 cm^2^/(V s))^42^ Additionally, the Type-II band alignment further contributes to lowering the recombination rate by spatially segregating electrons and holes in the individual monolayers. High carrier mobilities facilitate the rapid transport of photoinduced electrons and holes to the material surface, facilitating their involvement in the redox process. Our calculations affirm that the ZrSSe/SnSSe heterostructure exhibits carrier mobility comparable to that of other heterostructures, underscoring its excellent and dependable mobility performance. Similar to the band gap, the carrier mobility values of 2D Janus materials typically fall between those of their parent materials due to their structural and chemical similarities. This substantial disparity in electron and hole mobility effectively inhibits the recombination of photoexcited carriers, thereby enhancing photocatalytic efficiency through enhanced spatial separation of photogenerated carriers in 2D Janus materials.

### 3.7 Gibbs Free Energy Change for OER

**Figure 10:**
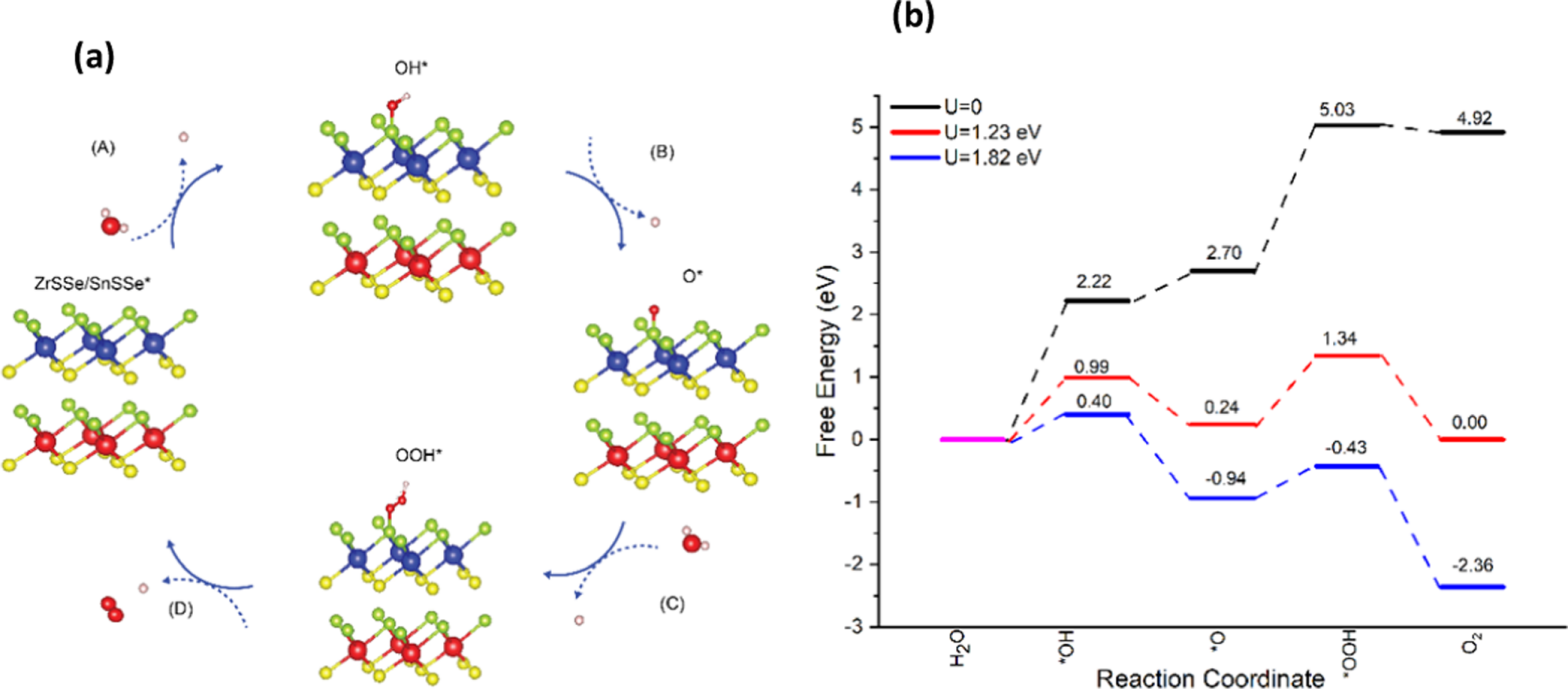
(a) Depiction of the energetically favorable OH*, O*, and OOH* intermediates in the four electron pathways of the OER on the ZrSSe/SnSSe heterostructure. (b) Computed Δ*G* values for the four electron pathways of the OER process at pH = 0.

To understand the OER performance of ZrSSe/SnSSe heterostructure, fully optimized adsorbed structures of the OH, O, and OOH with heterostructure (Figure 10(a)) were used and their Gibbs free energies were calculated with following steps.

The four electron transfer steps for the OER process are given by
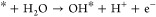
12
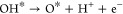
13
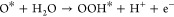
14
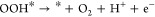
15

The Gibbs free energy change for each above step can be calculated as
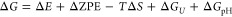
16Here Δ*E*, ΔZPE, Δ*S* represent the energy difference of the adsorption, corresponding changes of the zero-point energy, and entropy of the adsorption, respectively. Δ*G_U_* = −*eU* where *U* is the potential under, SHE (standard hydrogen electrode) and *e* is charge of electron, and Δ*G*_pH_ = 2.303*k*_B_*T* × pH.

Figure 10(b) displays the calculated Δ*G*s for the intermediate OER products, namely, O*, OH*, and OOH*. At a potential of *U* = 0 V (in the absence of light), we observed Δ*G* values for each OER step as follows: 2.22, 0.48, 2.33, and −0.11 eV for the heterostructure and these values are in the range of other 2D materials.^43^ These values indicate that every elementary step in the reaction requires an input of energy, except for the final step, which suggests a relatively weak interaction between the active sites and intermediates. Notably, the third step (O* → HOO*) is the rate-limiting step with an energy input of 2.33 eV. Consequently, this reaction is characterized as endothermic and nonspontaneous at zero potential, resulting in the OER process being hindered at different stages due to the increase in Gibbs free energy. The first step energy for water molecule dissociation is similar to energy 2.28 eV obtained in the case g-C_3_N_4_^44^

The overpotential for the OER reaction can be calculated by the following equation
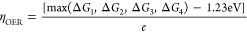
17Where Δ*G*_1_ = *G*_OH_, Δ*G*_2_ = *G*_O_ – *G*_OH_, Δ*G*_3_ = *G*_OOH_ – *G*_O_ and Δ*G*_4_ = 4.92 – *G*_OOH_

The calculated value of the overpotential at potential for *U* = 0 V (in the absence of light) of ZrSSe/SnSSe heterostructure is 1.1 V, which is similar to other 2D materials such as ScP (1.37 V), ScAs (1.18 V) and ScSb (1.02 V).^43−45^ The free energy diagram of the ZrSSe/SnSSe heterostructure at equilibrium potential *U* = 1.23 eV is also shown in Figure 10(b). It can be observed these elementary OER reaction steps become energetically favorable, except for the initial and third steps. In the presence of light irradiation, photogenerated holes provide an external potential of *U*_h_ = 1.82 eV at pH = 0, defined as the energy difference between the hydrogen reaction potential and the valence band maximum (VBM). Consequently, the Gibbs free energy profiles for the OER exhibit favorable downhill trends, except for the case where OH* reacts with another H_2_O molecule to generate the O* species. As a result, the limiting potential required to oxidize OH* species into O* species in a dark environment is 2.22 eV, but this value decreases to 0.40 eV under light irradiation.

As U_h_ are treated as the electrode potential relative to the SHE, this potential changes with the pH according to *U*_h_ = 1.82–0.059 × pH = 1.4 eV, which provides the external potential for the neutral environment.^45−47^ This shows that the external potential is reduced to 1.4 eV (pH 7) from 1.82 eV (pH 0); therefore, applying an external potential of 1.4 eV can start the OER in a neutral medium.

These findings suggest that the ZrSSe/SnSSe heterostructure may serve as a promising candidate for the OER under illuminated conditions.

### 3.8 Optical Properties

The optical properties of the ZrSSe/SnSSe heterostructure are also investigated using the HSE06 hybrid functional. The light absorption coefficient is obtained from the following formula
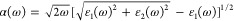
18where ω represents the frequency of incident light, ε_1_(ω)and ε_2_(ω) are real and imaginary parts of the dielectric constant, respectively.

**Figure 11:**
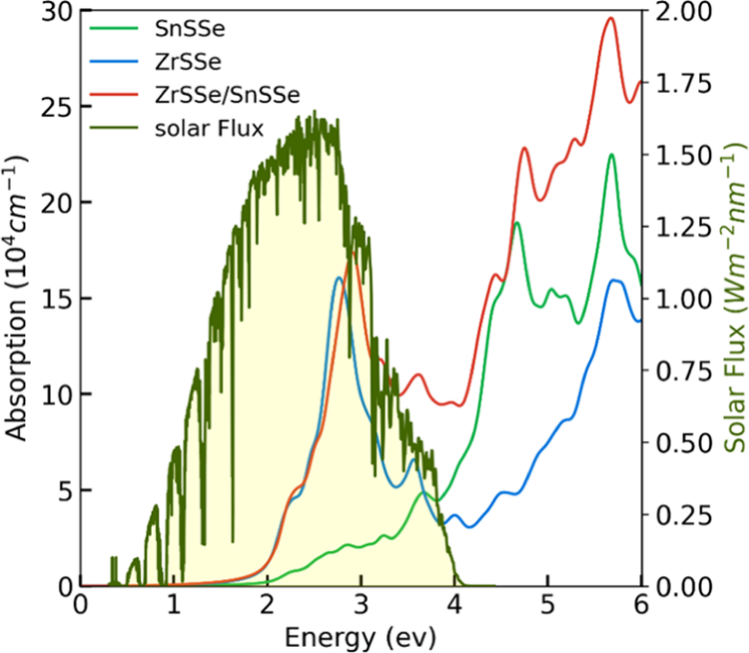
Absorption spectra of the heterostructure and monolayers.

To calculate the optical absorbance, we applied eq 18 to compute the optical absorption coefficients of the ZrSSe/SnSSe heterostructure and compared them with those of the ZrSSe and SnSSe monolayers, as depicted in Figure 11. From the graph, it is evident that the optical absorption spectrum of the ZrSSe monolayer exhibits a sharp absorption peak within the visible region (within the solar flux range), along with several absorption peaks in the ultraviolet region. In contrast, the SnSSe monolayer displays less pronounced and broader absorption features in both the visible and ultraviolet regions.^21,22^

Regarding the ZrSSe/SnSSe heterostructure, it is noteworthy that compared to the monolayers, the heterostructure demonstrates broader and more extensive light absorption across a significant portion of the solar flux spectrum. Additionally, the absorption spectra of the heterostructure reveal a substantial increase in the absorption intensity, primarily attributed to a reduction in the band gap. This observation underscores the superior light utilization capabilities of the heterostructure in both the ultraviolet and visible regions compared with the monolayers. The broader light absorption is attributed to charge transfer and interlayer coupling. The optical absorption spectrum confirms that the incorporation of a heterostructure enhances both the efficiency of visible light absorption and overall light absorption performance compared to monolayers.

Due to its type-II band alignment, the ZrSSe/SnSSe heterostructure readily facilitates the separation of photogenerated electron–hole pairs. This characteristic suggests that the ZrSSe/SnSSe heterostructure holds significant potential for applications in photocatalysis, solar cells, and various other photoelectric devices.
